# Supplementary material for: Fungal Species and Mycotoxins Associated with Maize Ear Rots Collected from the Eastern Cape in South Africa
Source: Toxins (Basel). 2024 Feb 8;16(2):95. doi: 10.3390/toxins16020095 (PMC10891880; doi:10.3390/toxins16020095)
Supplement: Supplementary file 1 [file toxins-16-00095-s001.zip › toxins-2811357-supplementary final/Supplementary/Supplementary Table S2 LC-MS MS method parameters Toxins January 2024.docx]

Supplementary Table S2. LC-MS/MS method parameters and method performance results.

| **Mycotoxin** | **Retention time, minutes** | **MRM Transitions ^a^** | | | **Recovery ^b^ %** | **Calibration curves ^c^** | | **Limit of quantitation (LOQ) ^d^, µg/kg** |
| --- | --- | --- | --- | --- | --- | --- | --- | --- |
|  |  | **Precursor ion, m/z** | **Product ion, m/z (Quantifier ion)** | **Product ion, m/z (Qualifier ion)** |  | **Concentration range of calibration curve, µg/kg** | **Calibration curve correlation coefficient, R^2^** |  |
| Aflatoxin B_1_ | 5.98 | 313.1 (M+H)^+^ | 285.1 | 241.1 | 87 | 1.25–80 | 0.996 | 2 |
| Aflatoxin B_2_ | 5.67 | 315.2 (M+H)^+^ | 287.2 | 259.1 | 90 | 1.25–80 | 0.992 | 2 |
| Aflatoxin G_1_ | 5.67 | 329.1 (M+H)^+^ | 243.1 | 283.1 | 93 | 1.25–80 | 0.990 | 2 |
| Aflatoxin G_2_ | 5.37 | 331 (M+H)^+^ | 245.1 | 257.1 | 90 | 1.25–80 | 0.983 | 5 |
| Deoxynivalenol | 0.87 | 297.2 (M+H)^+^ | 249.1 | 231.1 | 91 | 40–8000 | 0.998 | 100 |
| 15-Acetyl-deoxynivalenol | 4.15 | 339.2 (M+H)^+^ | 261.1 | 279.1 | 82 | 40–4000 | 0.994 | 100 |
| Diplodiatoxin | 7.74 | 309.40 (M+H)^+^ | 263.2 | 201.1 | 76 | 40-8000 | 0.995 | 50 |
| Fumonisin B_1_ | 6.32 | 722.4 (M+H)^+^ | 334.3 | 352.3 | 90 | 10–4000 | 0.995 | 20 |
| Fumonisin B_2_ | 7.09 | 706.4 (M+H)^+^ | 336.4 | 318.3 | 84 | 10–4016 | 0.996 | 20 |
| Fumonisin B_3_ | 6.78 | 706.4 (M+H)^+^ | 336.5 | 318.2 | 79 | 10–4048 | 0.997 | 20 |
| Ochratoxin A | 7.79 | 404.1 (M+H)^+^ | 239.1 | 358.1 | 69 | 1.25–80 | 0.997 | 2 |
| T2-Toxin | 7.43 | 484 (M+NH_4_)^+^ | 305 | 215 | 107 | 10–4000 | 0.991 | 20 |
| HT-2 toxin | 6.51 | 442 (M+NH_4_)^+^ | 263 | 215 | 119 | 10–4000 | 0.990 | 20 |
| Zearalenone | 7.73 | 319.2 (M+H)^+^ | 187.1 | 185.1 | 68 | 10–4000 | 0.991 | 20 |

^a^ MRM transitions on tandem quadrupole mass spectrometer (Waters AcquityTQD) ^b^ Percentage recovery of spiked sample at 50 ug/kg measured in batch of samples. Mycotoxin results not corrected for % recovery. ^c^ Calibration curves of matrix-matched standards prepared and analysed with the samples. Curves compiled with at least six concentration levels. Linearity of the curves were confirmed with the calibration curve correlation coefficient, R^2^. ^d^ Limit of quantitation (LOQ) (determined when the method was validated) is the lowest concentration level that can be quantified with acceptable precision and accuracy by applying the complete analytical method [33].
